# The application of barbed suture during the partial nephrectomy may modify perioperative results: a systematic review and meta-analysis

**DOI:** 10.1186/s12894-018-0435-3

**Published:** 2019-01-10

**Authors:** Yifei Lin, Banghua Liao, Sike Lai, Jin Huang, Liang Du, Kunjie Wang, Hong Li

**Affiliations:** 1 Department of Urology, Institute of Urology (Laboratory of Reconstructive Urology), West China Hospital, Sichuan University, Chengdu, Sichuan People’s Republic of China; 2 West China School of Medicine, Sichuan University, Guoxuexiang 37, Chengdu, 610041 Sichuan China; 3 West China Hospital, Sichuan University, Guoxuexiang 37, Chengdu, 610041 Sichuan China

**Keywords:** Partial nephrectomy, Surgical technique, Systematic review

## Abstract

**Background:**

Barbed sutures can avoid knot tying and speed the suture placement in the PN(partial nephrectomy). On account of the impact on clinical outcomes are ambiguous, this study is determined to identify the application of barbed suture during PN.

**Methods:**

ClinicalTrials.gov, Cochrane Register of Clinical Studies, PubMed and EMBASE were searched for RCTs(randomized controlled trials) and cohort studies focusing on the comparison of barbed and traditional sutures in PN(last updated on Feb in 2015). According to Cochrane Library’s suggestion, quality assessment was performed. Review Manager was applied to analyze all the data and sensitivity analyses were performed through omitting each study sequentially.

**Results:**

Eight cohort studies and none of RCTs proved eligible (risk of bias: moderate to low,431 patients). Warm ischemia time(MD = − 6.55,95% CI -8.86 to − 4.24, *P* < 0.05) decreased statistically in the barbed suture group, as well as operative time(MD = − 11.29,95% CI -17.87 to-4.71, *P* < 0.05). Postoperative complications also reduced significantly(OR = 0.44, 95% CI 0.24 to0.80, *P* < 0.05). Unidirectional barbed suture resulted in fewer postoperative complications based on the subgroup analysis(OR = 0.48,95% CI 0.24 to 0.94, *P* < 0.05).

**Conclusions:**

The barbed suture may be a useful surgical innovation which can modify perioperative results for surgeons and patients. Randomly-designed studies with longer follow up and larger sample sizes are in the need of to explore the applicability.

**Electronic supplementary material:**

The online version of this article (10.1186/s12894-018-0435-3) contains supplementary material, which is available to authorized users.

## Background

Partial nephrectomy (PN) is regarded as the golden standard treatment for small-localized renal tumors currently [1]. With the development of the surgical technology, larger (until 7 cm and more) cases may also be the appropriate candidates. Type of resection, type of suture and the change of renal function usually play a significant role during the perioperative period of PN [2]. Generally, warm ischemia time (WIT), among many factors to predict renal function, is the only predictor that can be modified by the surgeon or surgical techniques [3–5]. Though several surgical and technological innovations have been invented to enhance the efficiency of renorrhaphy and decrease WIT, such as the application of hemostatic agents, or even the involvement of the robot assistance [6, 7], different approaches still had their own advantages and disadvantages.

Recently, an innovative of absorbable, the knotless barbed suture is applied for renal pelvicaliceal or/and parenchymal repair to diminish WIT in laparoscopic partial nephrectomy [8]. Initiated in 1964 [9], this approach has been first reported in gynaecological and plastic surgery previously [10]. And then some systematic reviews, in terms of radical prostatectomy [11–13], have confirmed equivalence of biocompatibility and tensile strength of knotless barbed suture compared to conventional sutures in urological field [14, 15].

Since partial nephrectomy was one of the earliest urological surgeries that adopted this advanced technology, various effects were reported. Nevertheless, the effect of length of intraoperative ischemia on renal function and life quality of patients after PN is a subject of significantly heated discussion. Thus, a meta-analysis and systematic review was carried out for a more validated result on the application of knotless barbed sutures in PN comparing with the conventional sutures.

## Methods

### Study selection

A comprehensive literature search, which addressed the topic of barbed suture in partial nephrectomy (“barbed” OR “knotless” AND “suturing” OR “suture”) was performed. The databases includede Cochrane Register of Clinical Studies, MEDLINE, EMBASE and clinical trials registered in Clinicaltrials.gov. Available studies from inception to Feb. 21st, 2015 were evaluated for inclusion.

Randomized controlled trials (RCTs) or observational controlled studies reporting comparative outcomes of patients underwent partial nephrectomy using conventional suture or barbed suture were considered for inclusion. Excluded studies included patients using other materials to compare with barbed suture like mesh or staple rather than continues smooth sutures. Studies, including reviews, abstracts, overlapped studies and those published in languages other than English, were also excluded.

### Data extraction and outcome measures

Search results were entered into a bibliographic software (EndNote X7) for further analysis. Two individual investigators (YL, SL) screened all titles and abstracts collected from the search strategy for relevance and full text review.. Extracted data included family name of the first author, publication year, original country, sample sizes, study design, and postoperative complications.

Warm ischemia time, operative time, estimated blood loss or change in hemoglobin level, perioperative blood transfusion, changes in renal function, hospital stay and postoperative complications were considered to be the main outcome measures for the meta-analysis. We also evaluated the heterogeneity of the outcomes to confirm the appropriateness of pooling studies.

### Outcome definition

Operative time was defined as the total time of surgery. Warm ischemia time was determined from the minute of hilar clamping until the moment of unclamping which was the main procedure of partial nephrectomy. Estimated blood loss, change in hemoglobin level and perioperative blood transfusion were defined as the loss of blood during the surgery and it was generally acquired from the surgeons’ operative reports or/and anesthesia records. After surgeries, postoperative complications of the suture and hospital stay were also recorded. We specially evaluated the postoperative complications based on the modified Clavien classification [16]. Renal function, including serum creatinine (sCr) and estimated glomerular filtration rate (eGFR), were measured using different measurement tools, for instance, CKD-Epi and RENAL nephrometry score system.

### Quality assessment

Two authors (YL, SL) independently assessed the quality of each included study. Discrepancies were resolved by discussion and a consensus decision. For RCTs, the Cochrane risk of bias tools were used. Observational studies were evaluated using the Newcastle-Ottawa Scale to assess the risk of bias [17]. We included several categories for cohort studies: ascertainment of partial nephrectomy, representativeness of the barbed suture cohort, ascertainment of exposure to barbed suture, selection of the non-exposed cohort, demonstration that outcome of interest (i.e. warm ischemia time) was not reported at the beginning of study, comparability of study controls for important factors (e.g. adequate adjustment for confounders or matching for important confounding factors), assessment of outcome (e.g. adjudication and blinding assessment), and completeness of the follow up.

### Statistical analysis

Results for each study were calculated using a fixed effects model. (random effects mode for high heterogeneity) The existence of statistical heterogeneity was evaluated through the χ^2^ test and I^2^ test. Pooled standardized mean difference (SMD) and mean difference (MD)are estimated to evaluate the continuous data, and the pooled odds ratios (ORs) were calculated for the evaluation of dichotomous data through Review Manager (Version 5.3). Sub group meta-analysis was performed based on different barbed suture (unidirectional and bidirectional barbed suture). Sensitivity analyses were also performed. *P* < 0.05 was considered significant.

## Results

### Study selection process and characteristics

**Fig. 1:**
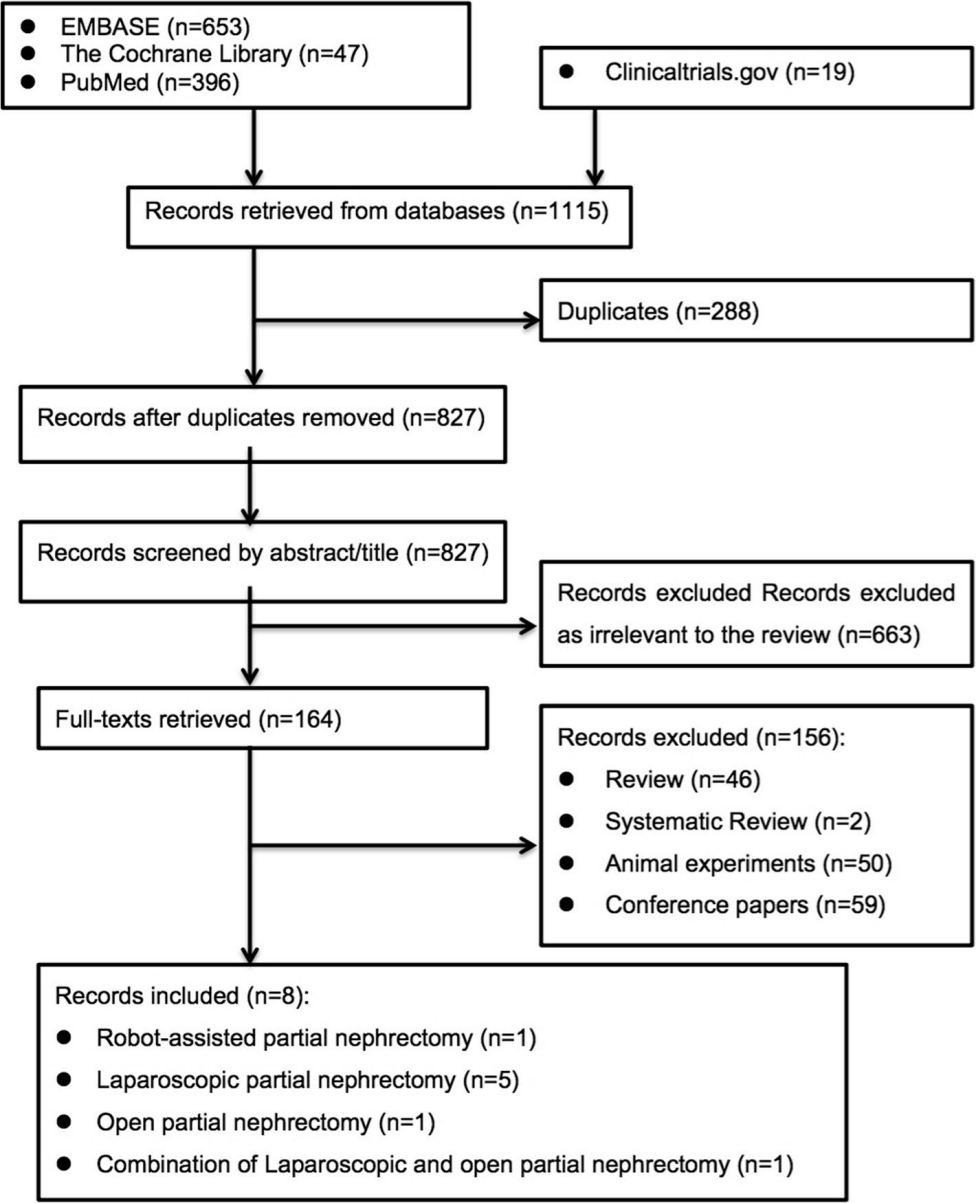
Flow diagram of the detailed selection process

A total of 8 cohort studies [18–25] (431 patients), and no RCTs proved eligible. A flow diagram of the detailed selection process is reported (Fig. 1). Table 1 shows the baseline characteristics and the results of all the eligible studies. All the data were comparable. Results of combined data comparing barbed suture versus conventional suture are presented in Table 2.

**Table 1:** Basic characteristics of all pooled studies in the meta-analysis

Author/year	Surgery type	Country	Barbed type	Study design	Renorrhaphy type	Median follow up	Sample size	Age	BMI	Tumor size	RENAL/ PAUDA/ Charlson Score	Suture time(min ± SD or range)	Warm ischemia time(min ± SD or range)	Operative time(min ± SD or range)	Estimated blood loss(ml ± SD or range/ mmol/L ± SD or range)	Perioperative Blood transfusion (n)	Hospital stay (days)	Postoperative complications
							B/C	B/C	B/C	B/C	B/C	B/C	B/C	B/C	B/C	B/C	B/C	
Sammon 2011	RAPN	US	U	Prospective cohort study	2 layers	1 month/4.5 months	15/15	64 ± 11.3/ 61 ± 8.4	29.8 ± 5.8/34.7 ± 7.2	2.5 ± 0.9/2.8 ± 1.4	NR	NR	18.5 ± 5.3/24.7 ± 6.6	227.5 ± 66.7/275 ± 49.2	150 ± 119.3/150 ± 99.6	NR	2.0 ± 0.7/2.0 ± 1.3	Bleeding
Olweny 2012	LPN	US	U	Prospective cohort study	Sliding clips	NR	29/49	55.1 ± 13.0/55.1 ± 12.9	30.4 ± 6.0/29.9 ± 6.3	2.6 ± 1.2/2.7 ± 1.2	NR	NR	26.4 ± 8.3/32.8 ± 7.9	174 ± 35/178 ± 41	188 ± 205/255 ± 337	1/5	3.4 ± 1.3/3.6 ± 0.98	Urine leak, embolization, other
Zondervan 2012	OPN& LPN	Netherlands	U	Prospective cohort study	1 layer	1 week	31/31	58 (27–80)/60 (25–81)	26.5 (20–39.2)/26.5 (17.0–47.9)	3.0 (1.6–7.1)/3.1 (1.5–6.8)	3 (0–9)/2 (0–9)¶	NR	19.6 ± 7.5/21.8 ± 9.5	NR	1.6 (0.0–3.8)/1.9 (0.3–9.2)	1/5	not stated/not stated	Bleeding,urinary fistula, lung embolism
Erdem 2013	LPN	Turkey	U	Retrospective cohort study	1 layer	NR	17/17	55 (29–80)/57 (32–78)	27.55 (19.53–31.25)/26.64 (21.25–30.43)	3.8 (2.2–6.3)/4.0 (2.0–6.5)	9 (6–13)/ 8 (6–12) §	NR	19 (12–50)/28 (10–57)	110 (90–190)/140 (80–190)	100 (30–400)/140 (20–200)	2/2	3 (2–5)/3 (2–7)	Fever, blood transfusion, elongated drainage, Ileus controlled with nasogastric decompression
Jeon 2013	LPN	US	U	Retrospective cohort study	Sliding clips	NR	13/24	60.1 ± 13.8/55.3 ± 11.7	28.3 ± 5.5/27.4 ± 7.3	2.5 ± 0.8/2.0 ± 0.7	7.1 ± 1.7/6.6 ± 1.6 *	NR	24.5 ± 5.3/31.9 ± 8.9	248.5 ± 37.2/256.7 ± 39.2	319.2 ± 311.3/331.3 ± 221.6	2/2	3.8 ± 1.3/3.8 ± 1.2	Postoperative hemorrhage, urine leakage, others
Shang 2013	LPN	China	Bi	Retrospective cohort study	2 layers	30 days	34/30	49 (25–83)/47 (19–74)	25.6/25.4	4/3.8	8.7 ± 1.0/ 8.6 ± 1.2*	NR	18/24.8	73 (45–135)/79.5 (40–135)	20 (5–600)/20 (5–350)	4/4	NR	Urinary fistula, extraction-site hernias, atelectasis, delayed hemorrhage
Schauer 2014	OPN	Austria	U	Retrospective cohort study	NR	NR	15/35	59.1 ± 13.7/65.5 ± 10.5	NR	3.5 ± 1.6/3.6 ± 1.2	9.0 ± 2.1/ 8.1 ± 1.8§	NR	NR	NR	15.6 ± 5.6/14.4 ± 8.9	NR	NR	Urinoma, pyrexia
Wang 2014	LPN	China	Bi	Retrospective cohort study	Sliding clips	1–11 months	36/40	51.3 ± 10.1/50.8 ± 11.2	NR	3.1 ± 1.2/3.0 ± 1.4	NR	10.4 ± 3.2 /19.4 ± 6.7	15.2 ± 4.2/24.1 ± 5.6	78.5 ± 15.4/90.3 ± 18.1	60.5 ± 21.2/110.4 ± 21.1	0/2	5.9 ± 2.1/6.8 ± 2.3	Blood urine, urine leak

*BMI* Body massive index, *US* the United States, *RAPN* Robot-assisted partial nephrectomy, *LPN* Laparoscopic partial nephrectomy, *OPN* Open partial nephrectomy, *U* Unidirectional barbed suture, *Bi* Bidirectional barbed suture, *B* Barbed suture group, *C* Control group, *NR* Not reported

*RENALScore

§PAUDA Score

¶Charlson Score

**Table 2:** Results of meta-analysis comparing barbed and control suture group

Outcomes of interest	Results of the combined studies						Study heterogeneity	
	Studies no.	Barbed group patients no.	Control group patient no.	SMD/MD/OR	95% CI	*P* value	I^2^	P value
Warm ischemia time	5	124	159	−6.55	−8.86 to −4.24	< 0.00001	50%	0.09
Warm ischemia time*	4	93	128	−7.85	−9.48 to-6.22	< 0.00001	0%	0.56
Operative time	4	93	128	−11.29	−17.87 to − 4.71	0.0008	17%	0.30
Estimated blood loss	5	108	163	0.50	−1.41 to 0.42,	0.29	91%	< 0.00001
Estimated blood loss*	4	72	123	−0.07	−0.36 to 0.23	0.66	0%	0.81
Perioperative blood transfusion	6	160	191	0.56	0.26 to 1.24	0.15	0%	0.58
Hospital stay	4	93	128	−0.21	−0.58 to 0.15	0.26	0%	0.49
Postoperative complications	8	190	241	0.44	0.24 to 0.80	0.007	0%	0.92

*CI* Confidence Interval, *SMD* Standardized Mean Deviation, *MD* Mean Deviation

*Sensitive analysis

Low to moderate risk of bias were considered among all the included studies (Additional file 1: Table S1). Specifically, the participant population within a study was identified from a same clinical setting comparing barbed suture with control. Ascertainment of patients performed partial nephrectomy was based on surgical records. A total of 3 studies have adjusted important confounding factors by matching pairs (body mass index and operative approach). No loss to follow-up in all studies.

### Warm ischemia time

**Fig. 2:**
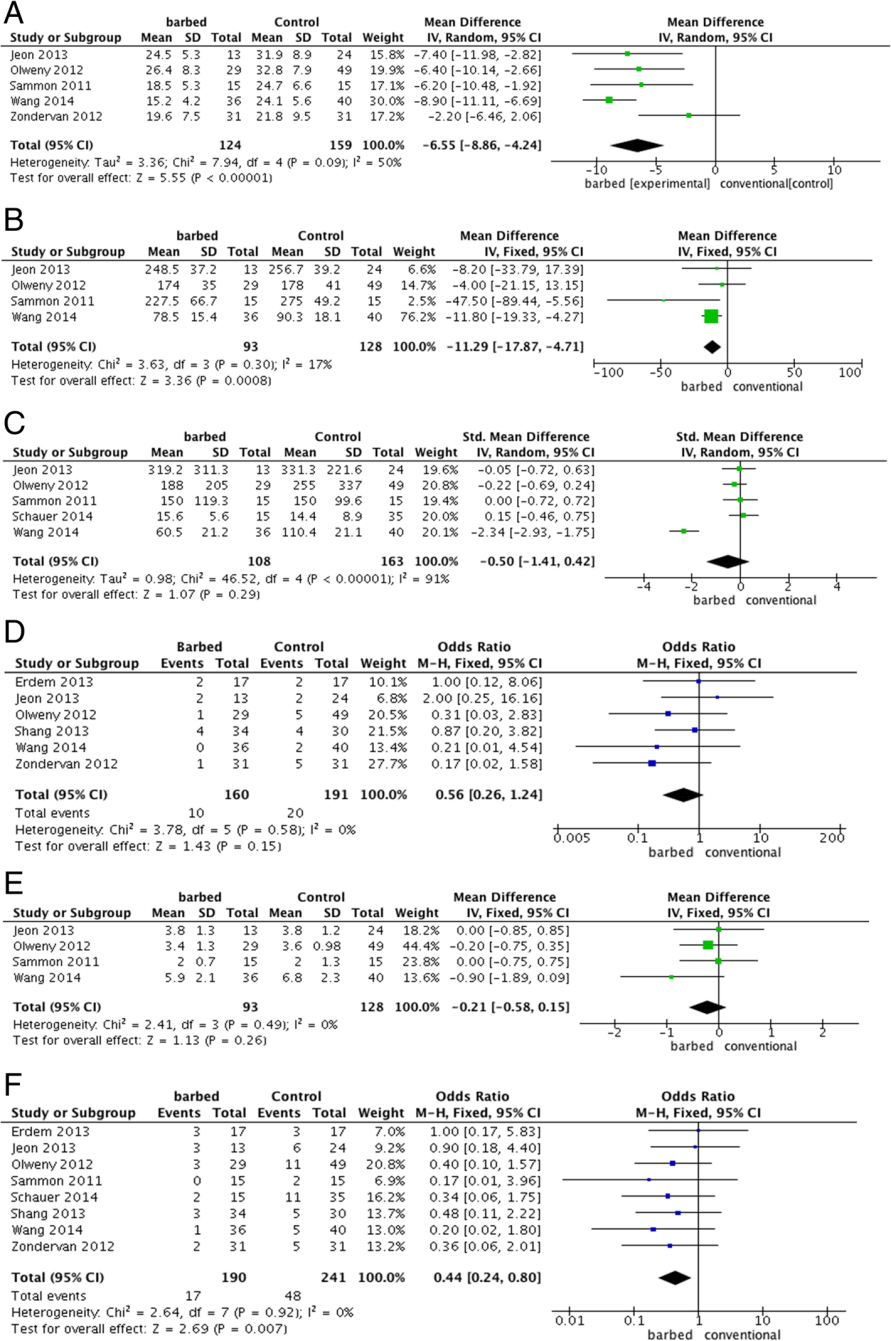
Pooled estimate of outcomes: (**a**) A forest plot of warm ischemia time with or without barbed suture; (**b**) A forest plot of operative time with or without barbed suture; (**c**) A forest plot of estimated blood loss with or without barbed suture; (**d**) A forest plot of perioperative blood transfusion with or without barbed suture; (**e**) A forest plot of hospital stay with or without barbed suture; (**f**) A forest plot of postoperative complications with or without barbed suture

Of the 8 cohort studies, 5 reported the warm ischemia time of partial nephrectomy [18–20, 22, 25] (Fig. 2a). Due to the fact that the heterogeneity is high among the WIT (*P* = 0.09, I^2^ = 50%), the random effects model was adopted instead of the fixed effects model. The pooling of raw data of these 283 cases suggested that patients with barbed suture versus conventional suture had a significantly shorter WIT. (MD = − 6.55, 95% CI -8.86 to − 4.24, *P* < 0.00001). Since Zondervan’s study recruited both laparoscopic and open surgeries whereas others only focused on laparoscopic partial nephrectomy, it was excluded for a sensitivity analysis. Therefore, the newly pooled results (221 cases) with low heterogeneity (I^2^ = 0%,) indicated that WIT in laparoscopic partial nephrectomy significantly decreased. (MD = − 7.85, 95% CI -9.48 to-6.22, *P* < 0.00001, Additional file 1: Figure S1).

### Operative time

Four studies of the laparoscopic partial nephrectomy reported data of operative time [18, 19, 22, 25] (Fig. 2b). The pooling of raw data presented a statistically significant association between the suture types (barbed sutures vs. conventional sutures) and operative time based on 221 cases (MD = − 11.29, *P* = 0.0008, 95% CI -17.87 to − 4.71) with low heterogeneity (I^2^ = 17%).

### Estimated blood loss

Five studies reported outcomes of estimated blood loss [18, 19, 22, 24, 25] (hemoglobin level or volume of blood loss). (Figure 2c) The pooling outcomes of the 271 cases did not present significantly benefits of barbed suture over conventional suture (SMD = − 0.50, 95% CI -1.41 to 0.42, *P* = 0.29). This finding was, however, highly limited due to a very high level of heterogeneity with random effect model (I^2^ = 91%).

Since Wang’s study [25] account for the bidirectional barbed suture (Quill SRS), while others applied unidirectional barbed suture (V-Loc™ 180), we therefore excluded this study for a sensitivity analysis. And the newly pooled results (195 cases) showed the same result while with low heterogeneity. (SMD = − 0.07, 95% CI -0.36 to 0.23, *P* = 0.66, I^2^ = 0%, Additional file 1: Figure S2).

### Perioperative blood transfusion

Six studies reported data of perioperative blood transfusion based on 351 participants. [19–23, 25] (Fig. 2d). Pooling of these cohort studies did not suggest a statistically significant association between the suture types and perioperative blood transfusion (OR = 0.56, 95% CI 0.26 to 1.24, *P* = 0.15, I^2^ = 0%). Moreover, subgroup analysis presented similar results, regardless of unidirectional or bidirectional barbed suture. (Additional file 1: Figure S3).

### Hospital stay

Four studies, with 221 patients recruited, reported about hospital stay [18, 19, 22, 25] (Fig. 2e). Pooling of those outcomes did not present a statistically significant association between the application of barbed suture and hospital duration. (MD = − 0.21, 95% CI -0.58 to 0.15, *P* = 0.26, I^2^ = 0%).

### Postoperative complications

Eight cohort studies reported raw event data postoperative complications [18–25] and the postoperative complications were evaluated based on the modified Clavien classification(Fig. 2f, Table 1) Pooling data of all the 431 cases showed that significantly fewer postoperative complications were found in patients with barbed suture versus conventional suture. (OR = 0.44, *P* = 0.007, 95% CI 0.24 to 0.80, I^2^ = 0%). Then the subgroup analyses (Additional file 1: Figure S4) by barbed suture types showed: compared with unidirectional barbed suture (V-Loc™), the use of barbed suture statistically decreases the postoperative complications. (OR = 0.48, 95% CI 0.24 to 0.94, *P* = 0.03, I^2^ = 0%); as for bidirectional barbed suture (Quill SRS), pooled data did not show significant differences between both groups (OR = 0.35, 95% CI 0.10 to 1.18, *P* = 0.09, I^2^ = 0%).

### Changes in renal function

As the reflection of renal function, sCr and/or eGFR were reported by four studies [20, 21, 23, 24] (Table 3). Measurement tools differed among four studies and only median and range data were reported. Though not readily for pooling, all did not suggest that significant changes between two suture groups in terms of both renal function indicators.

**Table 3:** Changes in renal function before and after PN (sCr & eGFR)

Author/year	Surgery	Cohort	N	Increase in sCr umol/L median [range]	P	Decline in eGFR ml/min/1.73 m^2^ median [range]	P	Measurement tools
Zondervan 2012	OPN & LPN	Barbed	31	10.0(− 70.7to114.0)	0.970	0.0(−4.3to14.2)	0.735	Creatinine was measured in the pre and postoperative period. Pre and postoperative estimated GFR were calculated using the CKD Epidemiology Collaboration (CKD-Epi) glomerular filtration equation
		Control	31	8.8(−26.5to123.8)		0.0(−4.6to18.6)		
Erdem 2013	LPN	Barbed	17	0(−0.3to2.3)	0.190	0(−23.0to39.0)	0.176	Functional renal preservation was assessed through the comparison of pre-operative and early postoperative eGFR, which was calculated using the Chronic Kidney Disease Epidemiology Collaboration formula. The early postoperative eGFR was based on an sCr measurement obtained after the peak sCr within the first 3 days of surgery.
		Control	17	0.1(−0.1to0.6)		9.0(−12to62)		
Shang* 2013	LPN	Barbed	34	2.59(−4.0 to 159.2)	0.797	7.7(−35.9to41.1)	0.065	RENAL nephrometry score system
		Control	30	2.65(−21.0 to 70.7)		8.9(−30.6to37.0)		
Schauer 2014	OPN	Barbed	15	20.1(−13.9to 51.9)	0.33	21.2(−18.0to38.0)	0.38	Postoperative changes in renal function parameters, serum creatinine levels, and eGFRs after surgery and before discharge.
		Control	35	10.3(−18.7to 61.5)		10.5(−26.1to42.5)		

*PN* partial nephrectomy, *LPN* laparoscopic partial nephrectomy, *OPN* open partial nephrectomy, *eGFR* estimated glomerular filtration rate, *sCr* serum creatinine

*The time point for renal function tests after surgery in this study was one month later after discharge from hospital, while the tests in other studies was right before the discharge from hospital

## Discussion

In this systematic review, we have included all the controlled studies to test the effects of barbed suture on PN. The pooling of all the cohort studies showed significant decreases in WIT, operative time, and postoperative complications in barbed suture group; the quality of included cohort studies was high to moderate. The subgroup analysis suggested that unidirectional barbed suture significantly minimized the postoperative complications, which seems to be safer than the traditional suture.

As the most important factor after partial nephrectomy, the pooled outcome of WIT witnessed an overall significant reduction in barbed suture groups, especially laparoscopic partial nephrectomies. This means barbed suture can effectively reduce the WIT during laparoscopic partial nephrectomies so as to improve the postoperative renal function.

SCr and eGFR were the most commonly used tools to evaluate renal function after PN [1], but the change of these two indexes were only reported in four studies [20, 21, 23, 24]. Since all the raw data suggested no significant shift between both groups, many researchers believed that the results needed larger sample size [24] and longer follow up time to confirm, say at least 5-year follow up [21]. Besides, there are many ways to determine loss of renal function and evaluate the effects of surgery on renal function, such as renal scan and volumetric assessment of the kidney, which were not reported in the articles included [26, 27]. Moreover, as the concentrations of them are enormously influenced by age, gender, muscle mass and so on [1, 3], a more convincing way to evaluate renal function loss in the patients, such as renal scintigraphy, needs to be focused on for future studies. Furthermore, the statistical decrease of operative time in barbed group also indicated that barbed suture was effective in partial nephrectomy either by a robot-assisted, laparoscopic, or open technique [28].

With regard to blood issue, both outcomes of estimated blood loss and transfusion were comparable. After performing the sensitivity analysis on the outcomes of estimated blood loss, the heterogeneity dropped from 91 to 0% with the pooled outcomes of still no significant difference. Similarly, the subgroup analysis of blood transfusion by different barbed suture did not suggest any difference from the overall effect. However, Zondervan et al [20]. reported that no difference was owing to the fact that hemostatic agents applied during surgery were not considered, thus this may be a confounding factor that need take into account in the future.

Meanwhile, number of postoperative complications was significantly minimized in barbed suture groups. Besides, subgroup analysis indicated that unidirectional barbed suture turned out to be safer than the convention suture while bidirectional barbed suture did not. However, no human study concerned the safety and efficacy among different barbed sutures. Thus the feasibility and safety among different barbed sutures in vivo study need to take into consideration [29, 30].

The pooled outcome in this study provides a reliable evidence for the relationship between the barbed suture and some important surgical indicators for PN. However, there are limitations of this study [1]. Even though two cohort studies adjusted their analyses via matching pairs, the pooled data may also be influenced by many factors, such as surgeons’ preference or learning curves due to none of RCTs recruited [2]. Because not all the studies evaluated the tumor via RENAL, PAUDA or Charlson scores before surgeries, it is impossible to perform subgroup analyses based on those indexes and future studies should uniform the standards to evaluate renal tumors [3]. The literatures search was extensive, but the barbed suture only suggested for all T1 kidney tumors rather than some suitable T2-may be or T3 cases and still did not involve animal studies, letters to the editors, and conference publications; and [4] because only 8 studies were recruited, the risk of publication bias could not be evaluated by the Begg’s funnel plots.

Nevertheless, our result renews a latest meta-analysis on barbed suture in PN. Based on what we know, this is to date the most comprehensive meta-analysis and systematic review exploring the relationship between barbed and traditional suture in PN.

## Conclusions

The barbed suture may be a useful surgical innovation which can modify perioperative results for surgeons and patients. Significant decline of suture time, operative time and postoperative complications were found using barbed suture during PN. Unidirectional barbed suture seemed to be safer. Because of the limitations of quality, especially that matched renal tumor complexity is not utilized, this state-of-the-art need to be proved by evidences with higher quality, and randomized-controlled studies with longer follow up and larger sample sizes will be needed to prove the findings of the present studies.

## Additional file

Additional file 1: **Figure S1.** A forest plot of sensitivity analysis of warm ischemia time with or without barbed suture. **Figure S2.** A forest plot of sensitivity analysis of Estimate blood loss with or without barbed suture. **Figure S3.** A forest plot of subgroup analysis of perioperative blood transfusion with or without barbed suture. **Figure S4.** A forest plot of subgroup analysis of postoperative complications with or without barbed suture. **Table S1.** Quality assessment of studies in the meta-analysis based on Newcastle-Ottawa Scale (NOS). (DOCX 92 kb)
