# Supplementary material for: The application of barbed suture during the partial nephrectomy may modify perioperative results: a systematic review and meta-analysis
Source: BMC Urol. 2019 Jan 10;19:5. doi: 10.1186/s12894-018-0435-3 (PMC6329109; doi:10.1186/s12894-018-0435-3)
Supplement: Supplementary file 1 — Figure S1. A forest plot of sensitivity analysis of warm ischemia time with or without barbed suture. Figure S2. A forest plot of sensitivity analysis of Estimate blood loss with or without barbed suture. Figure S3. A forest plot of subgroup analysis of perioperative blood transfusion with or without barbed suture. Figure S4. A forest plot of subgroup analysis of postoperative complications with or without barbed suture. Table S1. Quality assessment of studies in the meta-analysis based on Newcastle-Ottawa Scale (NOS). (DOCX 92 kb) [file 12894_2018_435_MOESM1_ESM.docx]

**Additional file Figure Legends**

Additional file 1: Figure S1: A forest plot of sensitivity analysis of warm ischemia time with or without barbed suture

Additional file 1: Figure S2: A forest plot of sensitivity analysis of Estimate blood loss with or without barbed suture

Additional file 1: Figure S3: A forest plot of subgroup analysis of perioperative blood transfusion with or without barbed suture

Additional file 1: Figure S4: A forest plot of subgroup analysis of postoperative complications with or without barbed suture

Additional file 1: Table S1: Quality assessment of studies in the meta-analysis based on Newcastle-Ottawa Scale (NOS)

Additional file 1: Figure S1: A forest plot of sensitivity analysis of warm ischemia time with or without barbed suture


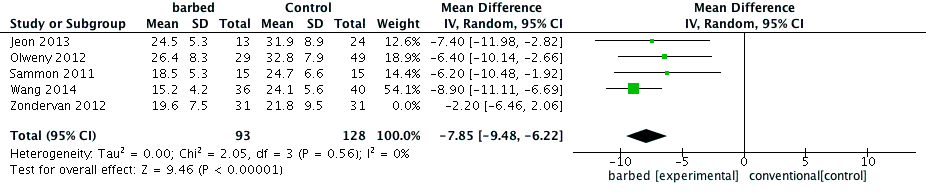


Additional file 1: Figure S2: A forest plot of sensitivity analysis of Estimate blood loss with or without barbed suture


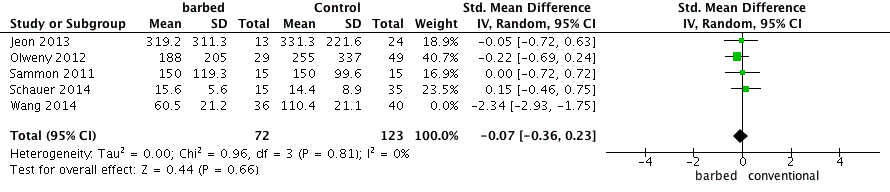


Additional file 1: Figure S3: A forest plot of subgroup analysis of perioperative blood transfusion with or without barbed suture


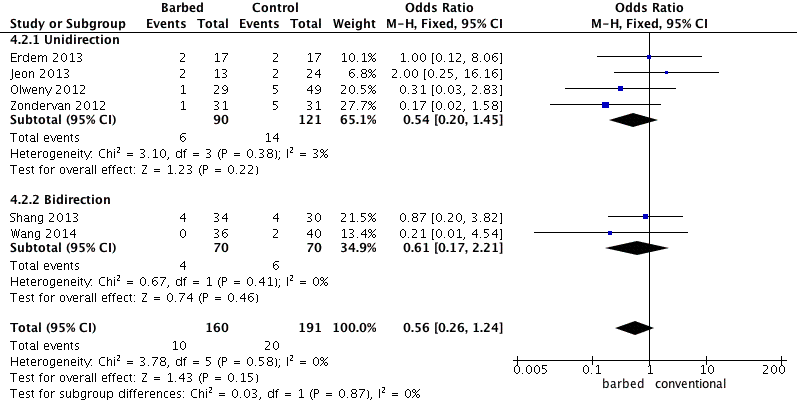


Additional file 1: Figure S4: A forest plot of subgroup analysis of postoperative complications with or without barbed suture


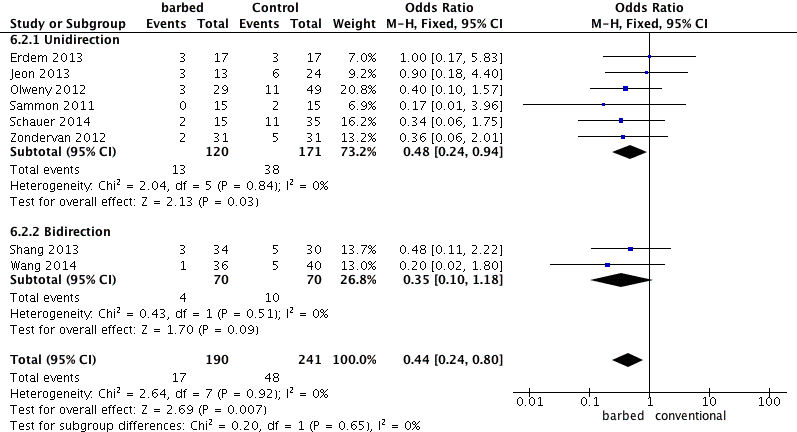


Additional file 1: Table S1: Quality assessment of studies in the meta-analysis based on Newcastle-Ottawa Scale (NOS)

Supplementary Table 1 Quality assessment of studies in the meta-analysis based on Newcastle-Ottawa Scale (NOS)

| **Author, year** | **Selection** | | | | | | | | **Comparability** | | **Outcome** | | | | | | **Total** |
| --- | --- | --- | --- | --- | --- | --- | --- | --- | --- | --- | --- | --- | --- | --- | --- | --- | --- |
|  | **1** | | **2** | | **3** | | **4** | | **1** | | **1** | | **2** | | **3** | |  |
| Sammon 2011 | A total of 30 consecutive patients underwent RAPN by a single experienced robotic surgeon | * | A total of 30 consecutive patients underwent RAPN by a single experienced robotic surgeon | * | Surgical records | * | Yes | * | No matching pairs, only statements of no differences between groups or that differences were not statistically significant and no adjustment in the analysis |  | Medical records | * | Yes | * | No lost of follow up | * | ******** |
| Olweny 2012 | Before July 2009, parenchymal repair after tumour excision was performed using absorbable polyglactin suture and subsequently, using SRBS | * | Before July 2009, parenchymal repair after tumour excision was performed using absorbable polyglactin suture and subsequently, using SRBS | * | Surgical records | * | Yes | * | No matching pairs, only statements of no differences between groups or that differences were not statistically significant and no adjustment in the analysis |  | Medical records | * | Yes | * | No lost of follow up | * | ******** |
| Zondervan 2012 | In both centers laparoscopic and open partial nephrectomy were performed according to standardized protocols. | * | In both centers laparoscopic and open partial nephrectomy were performed according to standardized protocols. | * | Surgical records | * | Yes | * | Propensity score matching was performed to create a 1:1 match of both groups based on three matching variables: PADUA score, surgical approach and center where the PN was done. No adjustment in the analysis | ** | Medical records | * | Yes | * | No lost of follow up | * | ********** |
| Erdem 2013 | Patients underwent LPN for suspected renal masses observed in radiologic evaluation. | * | Patients underwent LPN for suspected renal masses observed in radiologic evaluation. | * | Surgical records | * | Yes | * | Furthermore, 17 patients from each group were matched at a 1:1 ratio with respect to sex, age (within 5 years), body mass index (BMI) (maximum range of 3 kg/m2 in the same World Health Organization-BMI classification), preoperative aspects and dimensions used for an anatomic (PADUA) classification scoring system, and operative approach to eliminate the effects of these variables on WIT. | ** | Medical records | * | Yes | * | No lost of followup | * | ********** |
| Jeon 2013 | 37 patients who underwent a LPN performed by a single surgeon (B.I.C.) between May 2007 and December 2011 were enrolled in this study.The patients were divided into two groups based on the renorrhaphy technique. | * | 37 patients who underwent a LPN performed by a single surgeon between May 2007 and December 2011were enrolled in this study. The patients were divided into two groups based on the renorrhaphy technique. | * | Surgical records | * | Yes | * | No matching pairs,only statements of no differences between groups or that differences were not statistically significant and no adjustment in the analysis |  | Medical records | * | Yes | * | No lost of followup | * | ******** |
| Shang 2013 | A total of 34 patients were enrolled into SRS group.who were performed LPN using the SRS to remove a complex renal mass rRENAL score ≥7)from 2010 to 2012. | * | a pair match comparison was conducted with historical LPN cases from March 2008 to March 20 1 0 in which conventional suture was used. | * | Surgical records | * | Yes | * | Cases were matched for RENAL score.no adjustment in the analysis | ** | Medical records | * | Yes | * | No lost of followup | * | ********** |
| Schauer 2014 | 50 consecutive patients who underwent open partial nephrectomy under cold ischemia for a unicentric renal tumor between 2010 and 2012.In 15 patients, V-Loc™ SRBS (Covidien, Mansfield, MA, USA) were used during parenchymal reconstruction of the kidney. Conventional sutures were used in the remaining 35 patients. | * | 50 consecutive patients who underwent open partial nephrectomy under cold ischemia for a unicentric renal tumor between 2010 and 2012.In 15 patients, V-Loc™ SRBS (Covidien, Mansfield, MA, USA) were used during parenchymal reconstruction of the kidney. Conventiona sutures were used in the remaining 35 patients. | * | Surgical records | * | Yes | * | no matching pairs,only statements of no differences between groups or that differences were not statistically significant and no adjustment in the analysis |  | Medical records | * | Yes | * | No lost of followup | * | ******** |
| Wang 2014 | A total of 76 patient records were reviewed; all patients were diagnosed with renal carcinoma by CT or MRI before operation, | * | A total of 76 patient records were reviewed; all patients were diagnosed with renal carcinoma by CT or MRI before operation, | * | Surgical records | * | Yes | * | No matching pairs,only statements of no differences between groups or that differences were not statistically significant and no adjustment in the analysis |  | Medical records | * | Yes | * | No lost of followup | * | ******** |

Selection:

1) Representativeness of the Exposed Cohort

2) Selection of the Non-Exposed Cohort

3) Ascertainment of Exposure

4) Demonstration That Outcome of Interest Was Not Present at Start of Study

Comparability:

1) Comparability of Cohorts on the Basis of the Design or Analysis

Outcome:

1) Assessment of Outcome

2) Was Follow-Up Long Enough for Outcomes to Occur

3) Adequacy of Follow Up of Cohorts
